# Supplementary material for: MALINC1 an Immune-Related Long Non-Coding RNA Associated with Early-Stage Breast Cancer Progression
Source: Cancers (Basel). 2022 Jun 7;14(12):2819. doi: 10.3390/cancers14122819 (PMC9221538; doi:10.3390/cancers14122819)
Supplement: Supplementary file 1 [file cancers-14-02819-s001.zip › Supplementary Table S3.pdf]

Table S3. List of primers used in RT-qPCR analysis

Transcript	Primer sequence	Amplicon
MALINC1	Fw5´-CTACTTGGCTTCTTTCCTGGC-3´<br>Rv5´-TCCACTCTTTGGAAAAGCCCA-3´	187 bp
MALAT1	Fw5´-CACCGAAGGCTTAAAGTAGGAC-3´<br>Rv5´-GCTGACACTTCTCTTGACCTTAG-3´	93 bp
MTRNR1	Fw5´-ACACATGCAAGCATCCC-3´<br>Rv5´-GGCTGGCACGAAATTGA-3´	205 bp
JUN	Fw5´-CACAGAGAGACAGACTTGAGAAC-3´<br>Rv5´-ACTTGGATACCCTTGGCTTTAG-3´	86 bp
FOS	Fw5´-GAAGACCGAGCCCTTTGAT-3´<br>Rv5´-CTGCATAGAAGGACCCAGATA-3´	110 bp
RNA18S	Fw5´-GTAACCCGTTGAACCCCATT-3´<br>Rv5´-CCATCCAATCGGTAGTAGCG-3´	151 bp
GAPDH	Fw5´-ACAACTTTGGTATCGTGGAAGG-3´<br>Rv5´-GCCATCACGCCACAGTTTC-3´	294 bp
