# Supplementary figures and images for: MALINC1 an Immune-Related Long Non-Coding RNA Associated with Early-Stage Breast Cancer Progression
Source: Cancers (Basel). 2022 Jun 7;14(12):2819. doi: 10.3390/cancers14122819 (PMC9221538; doi:10.3390/cancers14122819)

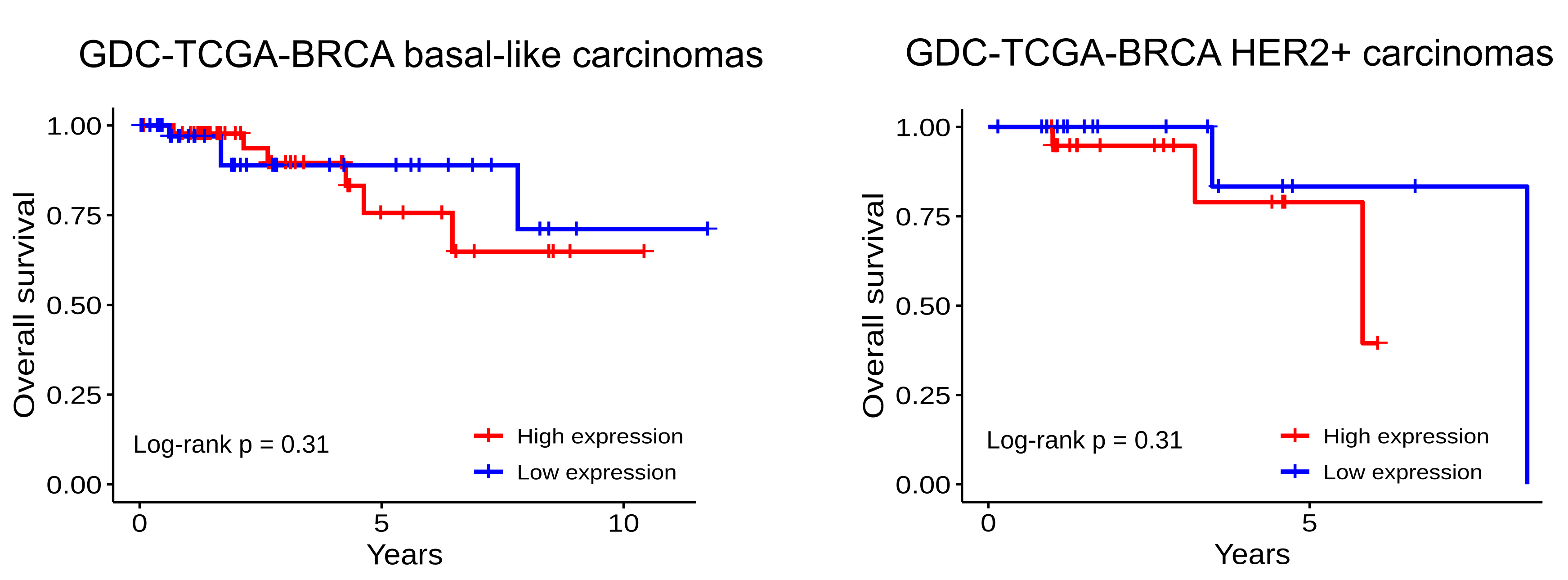

Supplement: Supplementary file 1 [file cancers-14-02819-s001.zip › Supplementary Figure S1.png]

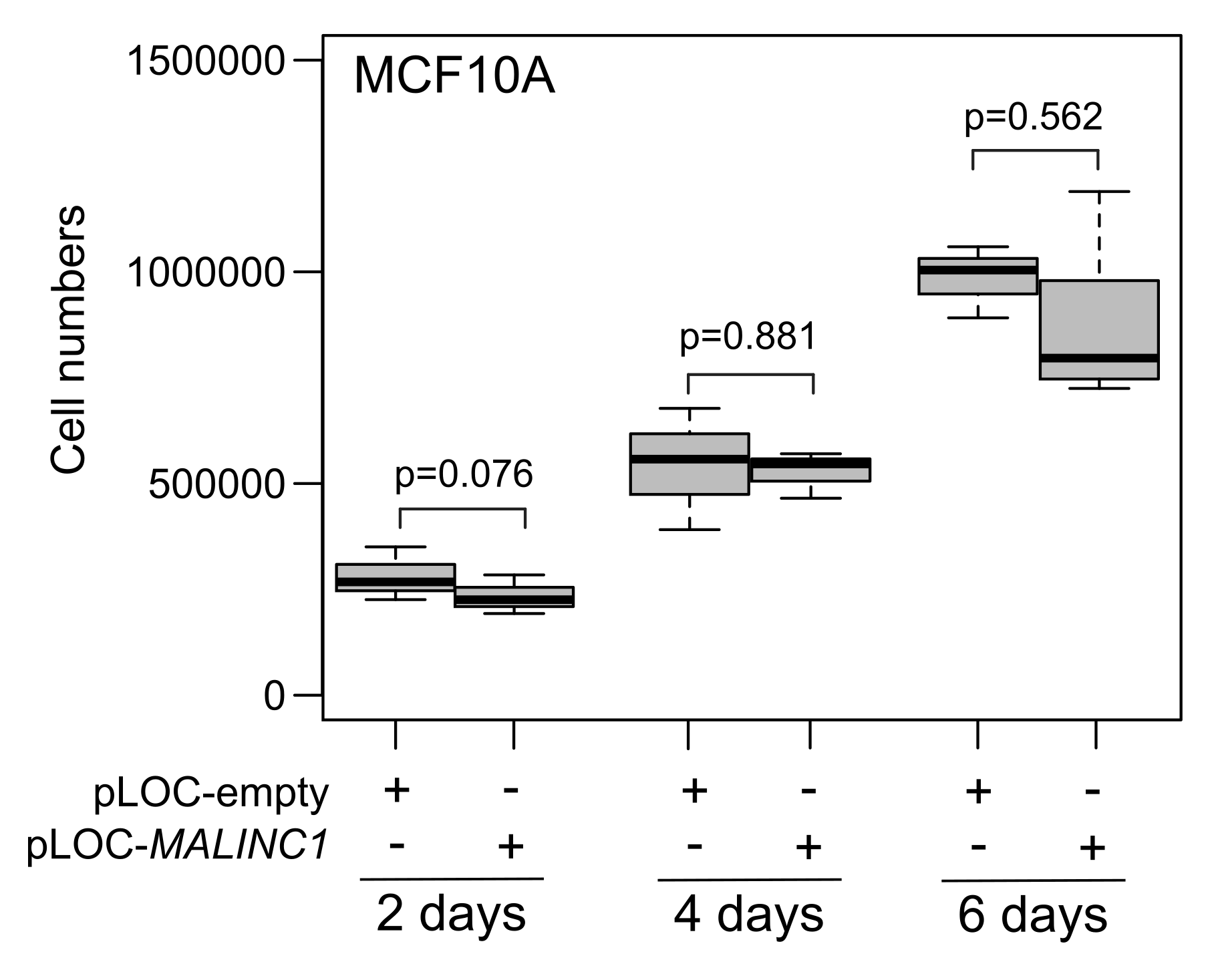

Supplement: Supplementary file 1 [file cancers-14-02819-s001.zip › Supplementary Figure S2.png]
